# Chromaturia and Providencia: A Case Report on Purple Urine Bag Syndrome

**DOI:** 10.7759/cureus.100997

**Published:** 2026-01-07

**Authors:** Hrithik Dakssesh Putta Nagarajan, PK Roshan, Md Ramij Biswas, Tejashvi Rameshkumar, Balakrishnan Kamaraj

**Affiliations:** 1 Department of Internal Medicine, Madurai Medical College, Madurai, IND; 2 Department of Emergency Medicine, Government Medical College, Thiruvananthapuram, Thiruvananthapuram, IND; 3 Department of Internal Medicine, Rajshree Medical Research Institute, Bareilly, IND; 4 Department of Internal Medicine, KAP Viswanatham Government Medical College, Tiruchirappalli, IND

**Keywords:** case report, providencia rettgeri, purple urine, purple urine bag, urinary tract infection

## Abstract

Purple urine bag syndrome (PUBS) is an uncommon condition typically observed in chronically ill, catheterized patients. This case report describes a 54-year-old Indian man with a history of recurrent strokes and long-term urinary catheterization who presented with lower abdominal discomfort and purple discoloration of the urine. Microbiological analysis revealed *Providencia rettgeri*, confirming the diagnosis of PUBS. The pathogenesis of PUBS involves the interaction between tryptophan and bacterial enzymes, resulting in the formation of colored compounds. Early identification of PUBS facilitates timely management, including treatment of the underlying urinary tract infection with appropriate antibiotics, catheter replacement, and addressing modifiable risk factors, which collectively may prevent potential complications and improve patient outcomes. This case highlights the importance of recognizing PUBS as a clinically relevant indicator of underlying urinary tract infections (UTIs) and emphasizes the need for enhanced care protocols in vulnerable populations. Increased awareness and expeditious management by health care providers can mitigate the impact of this condition.

## Introduction

Purple urine bag syndrome (PUBS) is an uncommon but visually distinct condition that typically manifests in chronically ill, bedridden, and debilitated patients. The most common risk factors for this condition include female sex and chronic urinary catheterization. It usually occurs secondary to urinary tract infections (UTIs) caused by specific bacterial infections that produce sulfatase or phosphatase enzymes. These enzymes facilitate tryptophan metabolism, resulting in the production of indigo (blue) and indirubin (red) pigments, which combine to create a purple coloration in alkaline urine [1,2]. This case report highlights the clinical presentation, diagnostic approach, and management of PUBS in a male patient with an underlying UTI caused by *Providencia rettgeri*.

## Case presentation

**Figure 1:**
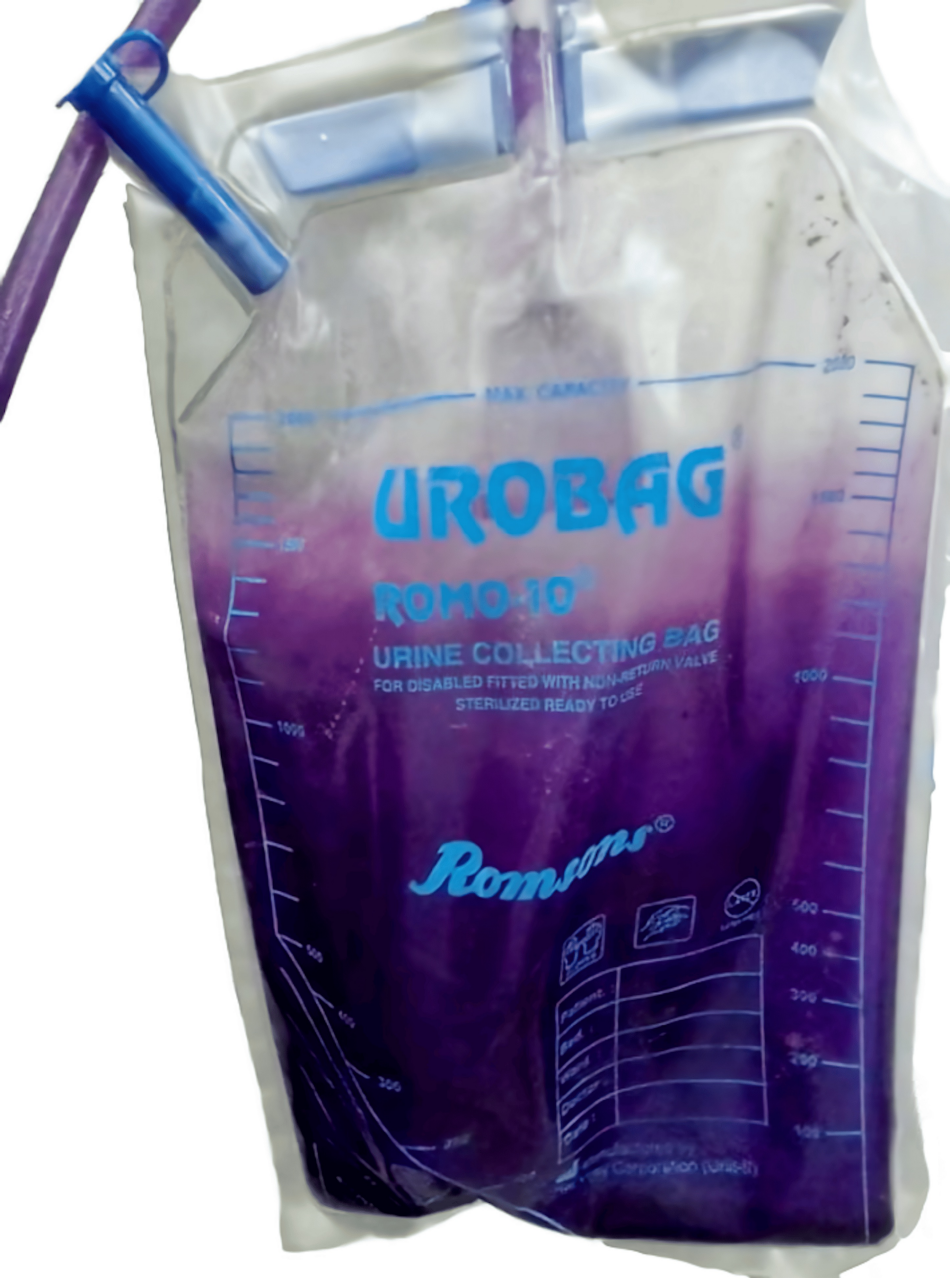
Urine bag of the chronically catheterized male with dark purple discoloration of urine.

A 54-year-old Indian male with a history of recurrent stroke (2009 and 2012) and a long-term urinary catheter in situ presented with symptoms of lower abdominal discomfort and a purple hue to the urine in the collection bag for the preceding month (Figure 1).

The patient also reported chronic constipation, for which he regularly used lactulose syrup. There was no reported history of fever, medication intake, use of food coloring, or ingestion of specific food items that could potentially alter the urine color. The urine exhibited a pungent ammoniacal odor.

A urine toxicology screen yielded negative results. A spot dipstick test revealed highly alkaline urine (pH 9) and was positive for leukocytes and nitrates, suggesting a urinary tract infection. PUBS was suspected at this juncture. Subsequently, an aliquot was dispatched to the Department of Microbiology for culture and sensitivity analysis. The patient was empirically treated with oral ciprofloxacin 1000 mg once daily, selected based on institutional antimicrobial guidelines for catheter-associated urinary tract infections, local resistance patterns, and preserved renal function. Concurrently, the existing urinary catheter was replaced with a new catheter. Microbiological analysis revealed substantial growth of a coliform species, identified as *Providencia rettgeri*, thereby confirming the diagnosis of PUBS. The organism demonstrated sensitivity to ceftriaxone.

The patient was given ceftriaxone 500 mg intravenously (IV) twice daily for 5 days. Subsequently, the urine color normalized, and repeat urinalysis yielded sterile results. The patient was then discharged. Upon telephonic follow-up one month after discharge, the patient reported no symptomatic complaints.

## Discussion

**Figure 2:**
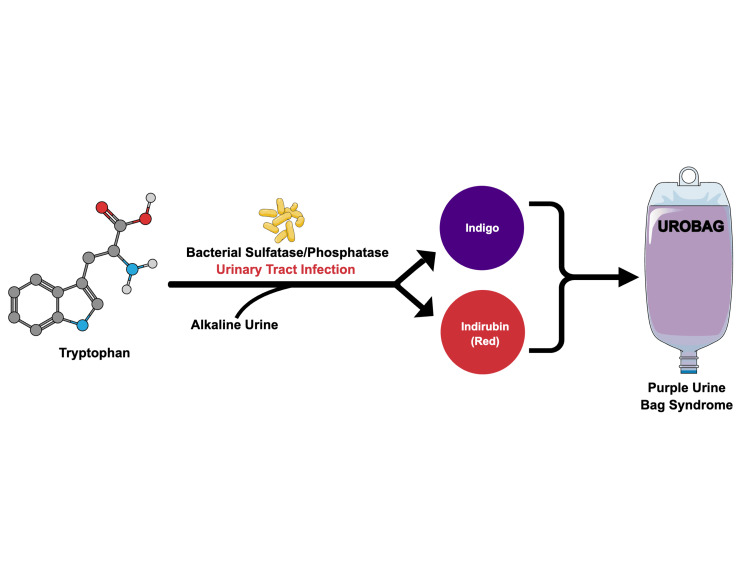
Simple graphical representation of the pathophysiology of PUBS. Figure created by the authors based on references [1-3]. PUBS: purple urine bag syndrome

**Figure 3:**
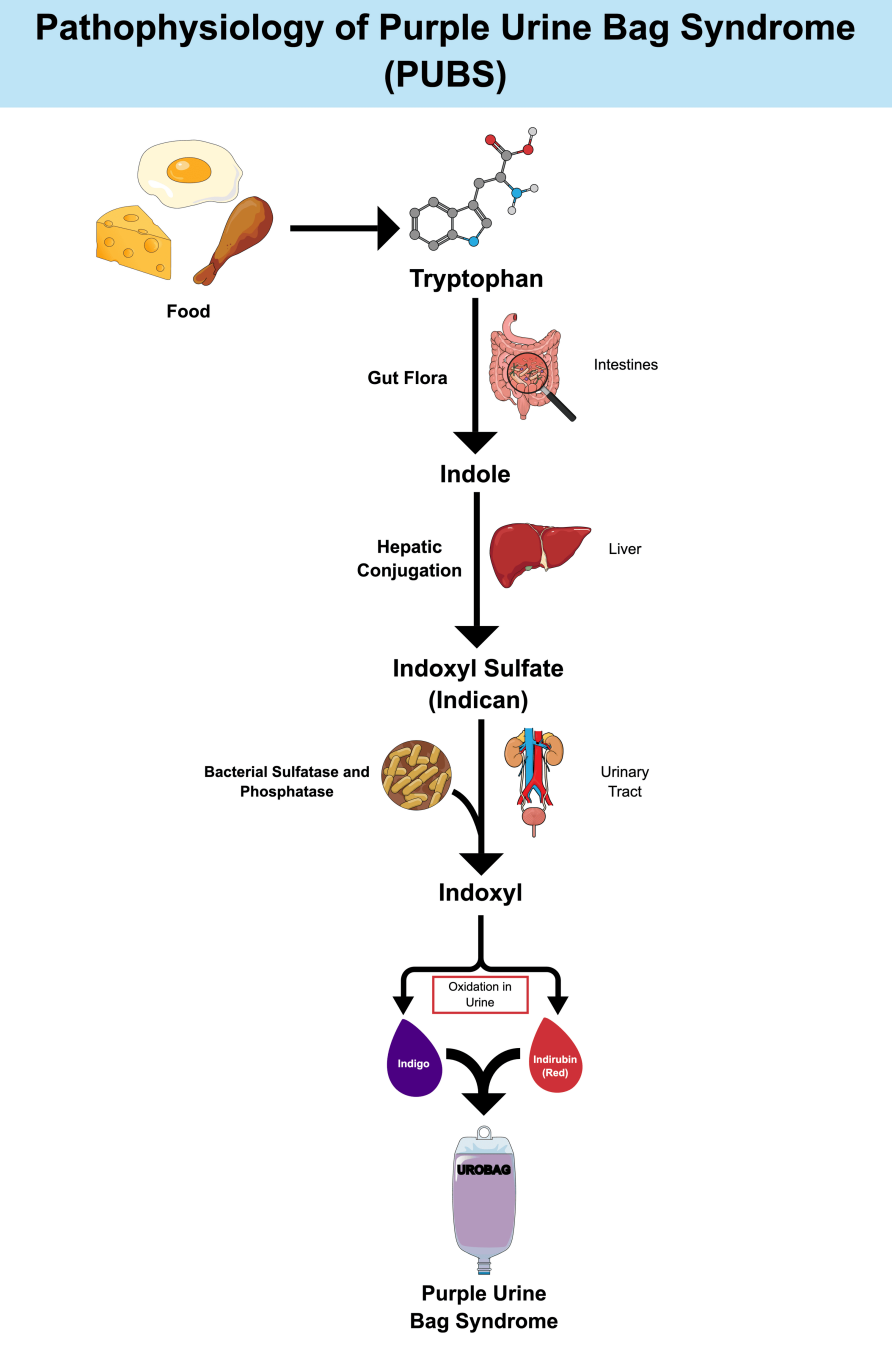
Detailed graphical representation of the pathogenesis of PUBS. Figure created by the authors based on references [1-3]. PUBS: purple urine bag syndrome

The pathogenesis of PUBS involves bacterial sulfatase and phosphatase enzymes in the urinary tract metabolizing tryptophan, resulting in the formation of indigo (blue) and indirubin (red) pigments that combine to create purple discoloration of the urine (Figures 2-3) [1-3].

**Figure 4:**
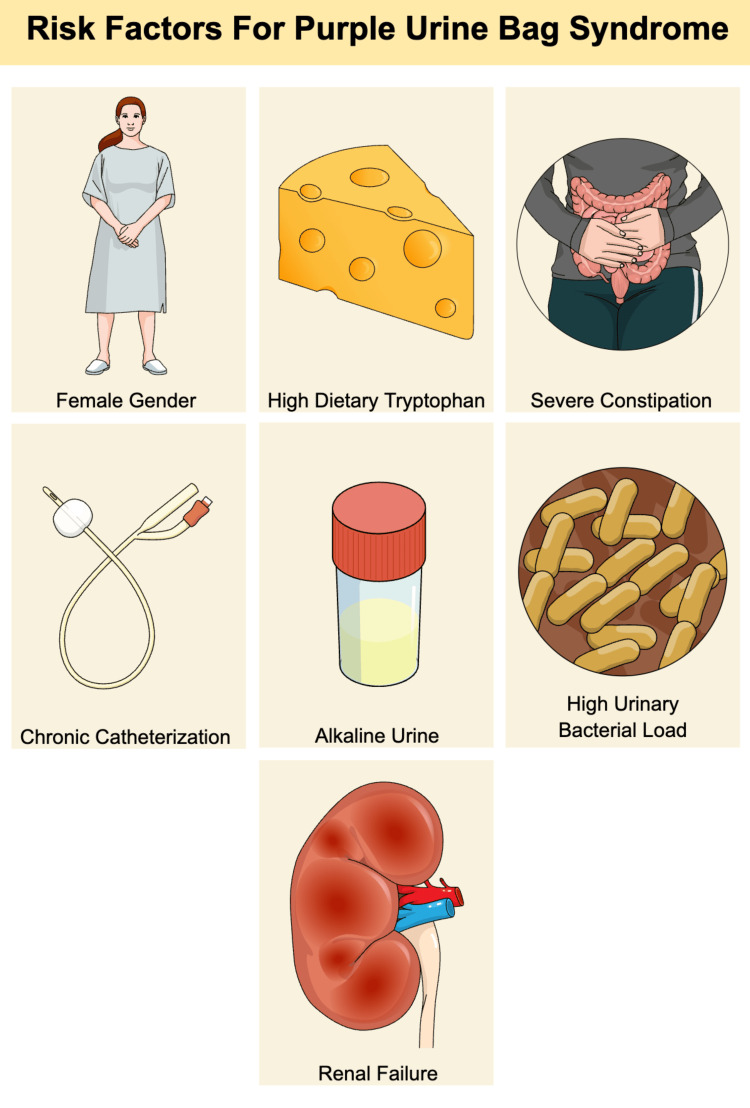
Risk factors for PUBS. Figure created by the authors based on reference [3]. PUBS: purple urine bag syndrome

Risk factors associated with the development of PUBS include female sex, alkaline urine, constipation, chronic catheterization, and renal failure (Figure 4) [3]. Although female sex is a recognized risk factor, our case demonstrates that PUBS can occur in male patients as well, highlighting the importance of considering this diagnosis regardless of gender when other risk factors are present.

The female population exhibits a higher susceptibility to urinary tract infections (UTIs) owing to their urogenital anatomy, consequently predisposing them to PUBS. Similarly, patients with chronic indwelling urinary catheters are at increased risk. Tryptophan serves as a substrate for the formation of indole and indirubin; thus, diets rich in tryptophan contribute to the development of PUBS [1,2]. Renal failure results in the impaired clearance of indoxyl sulfate, an intermediate in the pathogenesis of PUBS. Increased urine alkalinity facilitates the oxidation of indoxyl [4]. However, PUBS is occasionally observed, even in acidic urine [5]. An elevated bacterial load in the urinary tract increases the availability of sulfatase and phosphatase. Severe constipation is associated with prolonged bacterial deamination [6,7]. Although the typical risk factors for PUBS have been extensively studied, atypical presentations have been reported. PUBS has been demonstrated to be associated with intestinal intussusception in the literature [8].

Early identification of this syndrome can facilitate the timely management of the underlying UTI with replacement of the catheter, addressing the predisposing conditions, and administration of empiric antibiotic therapy, primarily targeting gram-negative organisms, particularly *Escherichia coli*, which has been identified as the most prevalent etiological agent of PUBS. The pathogen identified in this case study, *Providencia rettgeri*, has been associated with approximately 8% of PUBS, according to the findings reported by Su et al. (Table 1). Moreover, their findings indicated that the mortality rate did not correlate with the specific bacterial species responsible for PUBS [9].

**Table 1:** Distribution pattern of bacterial species associated with purple urine bag syndrome (PUBS) Findings reported by Su et al. [9]

Pathogen	Percentage of Cases
Escherichia coli	28%
Mixed microorganisms	18%
Enterococcus faecalis	13%
Proteus spp.	9%
Morganella morganii	9%
Klebsiella spp.	9%
Providencia rettgeri	8%
Pseudomonas aeruginosa	6%
Streptococcus spp.	2%
Staphylococcus spp.	1%

PUBS is predominantly a benign condition [10]; however, if left untreated, it may progress to adverse outcomes, with a reported mortality rate of up to 7% in certain studies. The factors that contribute to increased mortality in PUBS include uremia, shock, and diabetes mellitus. A modest increase in mortality has also been observed in female patients and individuals with leukocytosis [9]. In immunocompromised patients, it can sometimes lead to Fournier’s gangrene [11].

Moreover, enhanced awareness of this condition facilitates the mitigation of underlying risk factors in high-risk patients, including the implementation of more frequent catheter exchanges, the establishment of appropriate bowel regimens, and the improvement of care protocols for the geriatric population.

## Conclusions

PUBS is an unusual condition with a particularly benign clinical course. However, the clinical severity also depends on the severity of the underlying urinary tract infection and the patient’s comorbidities. Timely diagnosis, corroborated by laboratory investigations and microbiological culture, can facilitate the effective treatment and prevention of potential complications, especially in immunocompromised patients. Furthermore, this case emphasizes the necessity for enhanced care protocols, including regular catheter maintenance and addressing modifiable risk factors, to improve the outcomes and quality of life in vulnerable populations. Awareness of PUBS among healthcare providers and family members is crucial for mitigating undue concerns and enhancing patient outcomes. Further research is warranted to explore prevention strategies and their long-term implications.
